# Using Electrolyte Free Water Balance to Rationalize and Treat Dysnatremias

**DOI:** 10.3389/fmed.2018.00103

**Published:** 2018-04-23

**Authors:** Sanjeev R. Shah, Gautam Bhave

**Affiliations:** ^1^Renal-Electrolyte and Hypertension Division, Perelman School of Medicine, University of Pennsylvania, Philadelphia, PA, United States; ^2^Division of Nephrology and Hypertension, Department of Medicine, Vanderbilt University Medical Center, Nashville, TN, United States

**Keywords:** sodium, dysnatremia, electrolyte free water, free water, tonicity, balance

## Abstract

Dysnatremias or abnormalities in plasma [Na^+^] are often termed disorders of water balance, an unclear physiologic concept often confused with changes in total fluid balance. However, most clinicians clearly recognize that hypertonic or hypotonic gains or losses alter plasma [Na^+^], while isotonic changes do not modify plasma [Na^+^]. This concept can be conceptualized as the electrolyte free water balance (EFWB), which defines the non-isotonic components of inputs and outputs to determine their effect on plasma [Na^+^]. EFWB is mathematically proportional to the rate of change in plasma [Na^+^] (dP_Na_/dt) and, therefore, is actively regulated to zero so that plasma [Na^+^] remains stable at its homeostatic set point. Dysnatremias are, therefore, disorders of EFWB and the relationship between EFWB and dP_Na_/dt provides a rationale for therapeutic strategies incorporating mass and volume balance. Herein, we leverage dP_Na_/dt as a desired rate of correction of plasma [Na^+^] to define a stepwise approach for the treatment of dysnatremias.

Disorders of plasma [Na^+^] or dysnatremias are pathophysiologically relevant because of their connection to cell volume homeostasis. Cells maintain their volume within a homeostatic window to optimize function, which is particularly relevant for cells of the central nervous system. Animal cells lacking cell walls cannot maintain a hydrostatic pressure across their plasma membranes. Therefore, the difference in osmotic pressure between the intracellular and extracellular environments is the primary determinant of water movement across the cell plasma membrane. Water passively moves into or out of cells to equalize the intracellular and extracellular osmotic pressures at steady state. Cells will swell or shrink when abruptly placed in a hypotonic or hypertonic environment. The concentration of effective osmoles present within the intracellular fluid and the extracellular milieu determines their respective osmotic pressures. Therefore, cells generate and/or transport osmotically effective osmoles between the intracellular and extracellular compartments to actively regulate transcellular osmotic pressure gradients (1). The foremost transport mechanism is the ubiquitous movement of K^+^ ions intracellularly and Na^+^ ions extracellularly *via* the energy consuming Na^+^-K^+^ ATPase. This transport establishes K^+^ and accompanying phosphate and other anions as the predominant intracellular effective osmoles and Na^+^ along with Cl^-^ as their extracellular counterparts (2). If we assume that plasma, interstitial, and intracellular fluid compartments are in osmotic equilibrium, then a simplified one total body water (TBW) compartment-two osmole (Na^+^ and K^+^) model can be defined. In this model, plasma [Na^+^]_pw_ (defined as Na^+^ concentration in plasma water), the main determinant of plasma tonicity, equals total body tonicity which is defined as total body Na^+^ and K^+^ dissolved in TBW (2–5):

(1) $$\text{Plasma }{\left[\text{N}{\text{a}}^{+}\right]}_{\text{pw}}=\frac{\text{Total Body Na}+\text{K}}{\text{Total Body Water}}$$

Two main lines of evidence have been proposed in support of the body water-two osmole model. First, classic work determined that the osmotic pressure of most tissues is equal to plasma osmotic pressure suggesting tonicity equalization between the plasma and tissue interstitial and intracellular compartments (6). Second, the work of Edelman demonstrated that exchangeable Na^+^ (Na_e_), K^+^ (K_e_), and TBW primarily determine plasma [Na^+^]_pw_ with a linear relationship (7):

(2) $$\text{Plasma }{\left[\text{N}{\text{a}}^{+}\right]}_{\text{pw}}=1.11*\frac{\text{N}{\text{a}}_{\text{e}}+{\text{K}}_{\text{e}}}{\text{TBW}}-25.6$$

while the relationship defined by Edelman is consistent with the body water-two ion model, there are obvious differences as the linear regression slope is slightly greater than 1 at 1.11 and the *y*-intercept is not zero, but -25.6. These differences are likely due to known oversimplifications of the body water-two osmole model.

First, non-Na^+^ and K^+^ effective osmoles contribute to body tonicity. The most obvious clinical example is hyperglycemic crises with the accumulation of glucose contributing to extracellular tonicity and leading to “translocational” hyponatremia due to water movement from cells to their hypertonic environment. But less well understood, cells generate, consume, and/or transport organic osmolytes, including amino acid derivatives, polyols, and methylamines to alter intracellular tonicity and, therefore, extracellular tonicity and plasma [Na^+^] (1).

A second shortcoming of the body water-two osmole model is the assumption of tonicity equilibrium between plasma and interstitial extracellular compartments. Similar to the well-known hypertonicity of the renal medulla, many connective tissues, such as bone, cartilage, and skin are also hypertonic to plasma (8–10). Recent work has clearly demonstrated that animals and humans placed on high Na^+^ diets will store Na^+^ in skin without significant alteration of plasma tonicity and [Na^+^]. In these situations, exchangeable Na^+^ increases without the expected increase in plasma [Na^+^] as much of the accrued Na^+^ raises interstitial tonicity, but leaves plasma tonicity relatively unchanged (10–15). Cells within these hypertonic interstitial compartments are in tonicity equilibrium with their environment and probably acquire K^+^ and generate organic osmolytes to combat cellular crenation (16, 17). The potential for tonicity disequilibrium between plasma and interstitium, but not between interstitium and cells, highlights the fact that both hydrostatic and osmotic pressures determine water movement between plasma and interstitium, while only the latter defines water flow between cells and their environment (2). Taken together, future models of body tonicity need to account for at least three body fluid compartments, namely plasma, interstitial, and intracellular.

The Edelman plasma [Na^+^] relationship is defined in terms of “exchangeable” Na^+^, K^+^, and water. “Exchangeable” refers to the pool of body Na^+^, K^+^, and water that are measured through exchange with an exogenously administered radioisotope of the respective ion or water within hours to 1–2 days (18). All body water and nearly all of total body K^+^ are exchangeable, but only about 70–80% of total body Na^+^ is exchangeable with the remaining Na^+^ residing primarily in anhydrous bone matrix (2, 19, 20). Whether the bone pool of non-exchangeable Na^+^ can accrue or release Na^+^ in response to pathophysiologic stimuli is unclear, but has the potential to alter the relationship between Na^+^ balance and plasma [Na^+^] (21–25).

As opposed to the body water-two osmole “idealized” plasma [Na^+^] relationship, the empiric Edelman relationship does potentially account for non-Na^+^ and K^+^ osmoles and Na^+^ storage in connective tissues in its “non-ideal” slope and *y*-intercept (26), but these values probably do not remain constant across physiologic states. Moreover, as the amount of Na^+^ stored in connective tissues and cellular osmolytes change during the genesis and treatment of dysnatremias, so too will the slope and *y*-intercept of the relationship. Edelman simply captured a cross-sectional average specific to his cohort of patients equilibrated to a low Na^+^ diet.

Chronic anti-diuretic hormone (ADH) treatment in animals and chronic SIADH in humans is an illustrative example of how complex alterations in body osmole homeostasis limits the quantitative application of any simple linear relationship with fixed slope and *y*-intercept between plasma [Na^+^] and exchangeable Na^+^, K^+^, and water. ADH action is initially thought to lower plasma [Na^+^] primarily through gain of water leading to expansion of cellular and extracellular volume. Extracellular volume expansion leads to negative Na^+^ balance to re-establish euvolemia, while cells jettison intracellular K^+^ and organic osmoles to lower cell volume. In the end, hyponatremia results from a combination of Na^+^, K^+^, and organic osmole losses and body water expansion (27, 28). Moreover, alterations in external Na^+^, K^+^, and water balance only partly explain ADH induced hyponatremia suggesting a significant role for non-Na^+^ and K^+^ osmoles and possible Na^+^ storage or release from connective tissues (29–32).

Given their theoretical limitations, it is not surprising that the idealized plasma [Na^+^] or the empiric Edelman relationship and equations derived from them only modestly predict alterations in plasma [Na^+^] in clinical situations, where external Na^+^, K^+^, and volume balances are equated to alterations in exchangeable Na^+^, K^+^, and water to calculate changes in plasma [Na^+^] (33–37). In addition to theoretical limitations, we are often not privy to a complete “balance sheet” of the volume and composition of inputs and outputs at the bedside. Some gains and losses are not measured even in the most rigorous balance studies, such as insensible losses and metabolic water production. Even when inputs and outputs and their composition are known, we assume the balance sheet remains constant, which is probably only true over very short time frames. For example, suppression of ADH secretion with volume resuscitation in hypovolemic hyponatremia may produce a brisk diuresis and an unintentional over-correction of plasma [Na^+^] (38). Given the modest predictability of plasma [Na^+^] equations, close clinical and laboratory follow-up with iterative alterations in the therapeutic plan are mandatory. While we recognize these shortcomings, we suggest that the foundation to understand the pathogenesis of dysnatremias and initiate rational therapies at the bedside currently rests on the relationship between plasma [Na^+^] and total body Na^+^, K^+^, and water until better alternatives are identified. In this review, we use the idealized plasma [Na^+^] relationship for clarity, but many of the mathematical relationships described may be modified using the empiric Edelman relationship as done by Nguyen and colleagues (39).

Even with the idealized plasma [Na^+^] relationship, a complex aspect of dysnatremias is that they involve the balance of Na^+^, K^+^, and water in relation to their relative proportions rather than the simple balance of any one species. But how can this balance and relative proportionality be quantified to more accurately reflect the physiology involved in the pathogenesis of dysnatremias? Herein, we draw attention to dysnatremias as disorders of electrolyte free water balance (EFWB). Intuitively, most clinicians recognize that isotonic gains or losses do not alter plasma [Na^+^], while hypo- or hypertonic changes alter plasma [Na^+^]. Indeed, we routinely administer isotonic NaCl to replenish extracellular volume without altering plasma [Na^+^] and use hypotonic D_5_W to expand TBW and lower plasma [Na^+^]. The conceptualization of inputs and outputs as part isotonic and part “electrolyte free water” with only the latter affecting plasma [Na^+^] concentration arises from the intuitive notion that only hypo- or hypertonic gains and losses affect plasma tonicity (4, 40, 41). Steady state plasma [Na^+^] is maintained when the difference in input and output electrolyte free water or EFWB equals zero, while persistently positive or negative EFWB lead to hyponatremia and hypernatremia, respectively (40, 42).

## EFWB is Related to the Rate of Change in Plasma [Na^+^]

Electrolyte free water balance is a volumetric rate, a change in volume per unit time, rather than a volume. Therefore, EFWB would be related, not to plasma [Na^+^], but to the *rate of change* in plasma [Na^+^] (dP_Na_/dt). Indeed, the instantaneous rate of change of plasma [Na^+^] (dP_Na_/dt) derived from the idealized plasma [Na^+^] relationship is proportional to EFWB (see Data Sheet S1 in Supplementary Material):

(3) $$\frac{\text{d}{\text{P}}_{\text{Na}}}{\text{dt}}=\frac{-\text{EFWB}}{\text{TBW}}*{\text{P}}_{\left[\text{Na}+\text{K}\right]}$$

where EFWB is the difference between electrolyte-free water input (EFWI) and electrolyte-free water clearance (EFWC):

(4) EFWB=EFWI−EFWC

**Figure 1:**
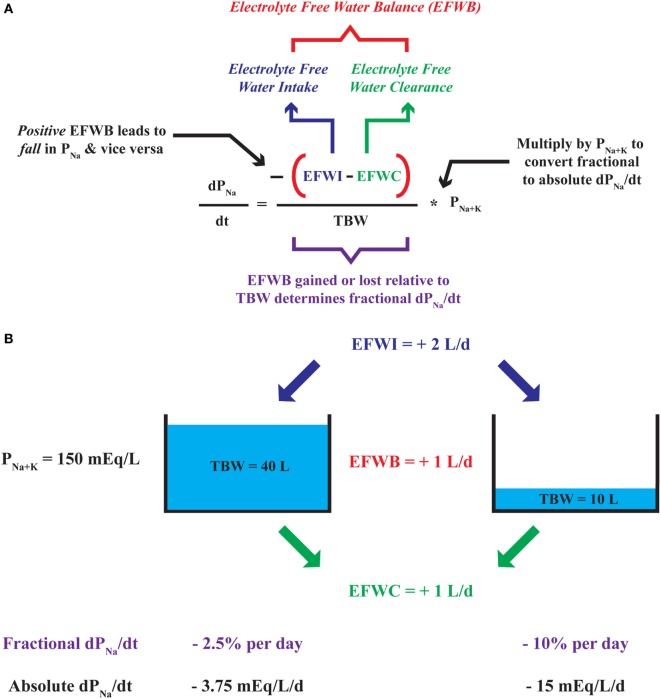
Deconstruction of the dP_Na_/dt—electrolyte-free water balance (EFWB) relationship. **(A)** The rate of change in plasma [Na^+^] or dP_Na_/dt is negatively proportional to EFWB such that positive EFWB lowers plasma [Na^+^] and negative EFWB raises plasma [Na^+^]. EFWB is divided by total body water (TBW) to yield fractional dP_Na_/dt and multiplied by P_[Na+K]_ to yield an absolute rate of change in plasma [Na^+^]. **(B)** Since TBW scales EFWB, a 1 L/day increase in EFWB leads to a larger fractional and absolute daily change in plasma [Na^+^] when TBW is comparatively small (10 versus 40 L).

Analogous to any balance which subtracts output from input, EFWB can be created by subtracting EFWC from EFWI (40, 42). The relationship corroborates our clinical intuition as a gain of electrolyte-free water or a positive EFWB yields a negative dP_Na_/dt and lowers plasma [Na^+^] and *vice versa*. An EFWB of zero or no net change in electrolyte-free water leads to a dP_Na_/dt equal to zero and no change in plasma [Na^+^]. Furthermore, we can use the dP_Na_/dt relationship to quantitatively predict changes in plasma [Na^+^] based on EFWB. Akin to adding or removing water from a “tank,” EFWB is scaled to TBW to yield a fractional (or percent) rate of change in plasma [Na^+^] (Figure 1). In effect, TBW “buffers” plasma [Na^+^] against the effect of each liter of electrolyte-free water gained or lost. Thus, elderly or malnourished patients with low TBW are at increased risk for the development of dysnatremias or iatrogenic complications of their overcorrection (38).

Like EFWB, EFWI, and EFWC are volumetric rates not volumes. Indeed, EFWC is designated as a “clearance,” a volumetric rate similar to creatinine clearance, which is expressed as mL per min or volume per unit time. The classic mathematical representation of EFWI and EFWC focuses on input or output tonicity relative to plasma tonicity:

(5) $$\begin{array}{llll} \text{EFWI} & ={\text{V}}_{\text{I}}*\left[1-\left({\text{I}}_{\left[\text{Na}+\text{K}\right]}∕{\text{P}}_{\left[\text{Na}+\text{K}\right]}\right)\right]\;\\ & \;\text{Relative Tonicity}\; & & \; \end{array}$$

(6) $$\begin{array}{llll} \text{EFWC} & ={\text{V}}_{\text{O}}*\left[1-\left({\text{O}}_{\left[\text{Na}+\text{K}\right]}∕{\text{P}}_{\left[\text{Na}+\text{K}\right]}\right)\right]\;\\ & \;\text{Relative Tonicity}\; & & \; \end{array}$$

where V_I_ and V_O_ are input and output flow rates and I_[Na+K]_ and O_[Na+K]_ are input and output Na + K concentrations, respectively. The relative tonicity term in Eqs 5 and 6 (in bold) is negative when the input or output is hypertonic (I_[Na+K]_ or O_[Na+K]_ > P_[Na+K]_) and, therefore, yields a negative value for EFWI and EFWC respectively. Conversely, the relative tonicity term is positive with hypotonic inputs or outputs (I_[Na+K]_ or O_[Na+K]_ < P_[Na+K]_) leading to a positive EFWI or EFWC, respectively. Finally, EFWI and EFWC equal zero with isotonic inputs or outputs, where I_[Na+K]_ or O_[Na+K]_ equal P_[Na+K]_. This mathematical construct aligns with clinical intuition. Isotonic inputs and outputs do not change plasma [Na^+^]. Hypertonic inputs with negative EFWI or hypotonic outputs with positive EFWC tilt EFWB toward a negative value and, therefore, tend to raise plasma [Na^+^]. Conversely, hypotonic inputs with positive EFWI or hypertonic outputs with negative EFWC push EFWB toward a positive value and tend to lower plasma [Na^+^].

But how do we appropriately define plasma [Na + K] to gage relative tonicity? Laboratories report plasma [Na + K] as molar concentrations expressed as mEq per L of plasma. However, osmolality and tonicity are directly proportional to the *molal* concentrations of dissolved solutes expressed as mEq per kg of *plasma water* (plasma [Na + K]_pw_) rather than the lab reported molar values. Therefore, plasma [Na + K]_pw_ is the appropriate reference to define relative tonicity of inputs and outputs to plasma when calculating EFWI and EFWC. Plasma [Na + K] and [Na + K]_pw_ differ because plasma is about 93% water with lipid and protein occupying most of the remaining volume (43, 44). Thus, we can convert reported molar plasma [Na + K] into molal plasma [Na + K]_pw_ using the fraction of plasma occupied by water (f_PW_, units of kg plasma water per L of plasma):

(7) $$\text{Plasma }{\left[\text{Na}+\text{K}\right]}_{\text{pw}}=\text{Plasma }\left[\text{Na}+\text{K}\right]∕{\text{f}}_{\text{PW}}$$

with a normal reported plasma [Na + K] = 144 mEq/L and f_PW_ = 0.93, normal plasma [Na + K]_pw_ = 154.8 mEq/kg H_2_O. Pseudohyponatremia exemplifies the direct relationship between molal plasma [Na + K]_pw_ and tonicity. In this condition, plasma [Na + K]_pw_ and tonicity are unchanged, but a fall in f_PW_ results in a low molar plasma [Na + K] as determined by the clinical laboratory. For example, if f_PW_ falls to 0.8, the laboratory reports a plasma [Na + K] equal to 123.8 mEq/L (154.8 mEq/kg H_2_O*0.8 kg H_2_O/L serum) even though plasma [Na + K]_pw_ and osmolality are unchanged. With plasma and extracellular tonicity unchanged, no specific action is required (45). In contrast to plasma, input and output [Na + K] molar concentrations nearly equal molal concentrations (within 1%), since these fluids are composed of relatively small amounts of solute dissolved in water with the possible exception of nephrotic urine. Therefore, 0.9% NaCl (normal saline) with a concentration of 154 mEq Na^+^ per L of solution is truly isotonic matching plasma [Na + K]_pw_. Based on molal plasma [Na + K]_pw_ as a gage of plasma tonicity, we better define EFWI and EFWC:

(8) $$\begin{array}{llll} \text{EFWI} & ={\text{V}}_{\text{I}}*\left[1-\left({\text{I}}_{\left[\text{Na}+\text{K}\right]}∕{\text{P}}_{\left[\text{Na}+\text{K}\right]\text{pw}}\right)\right]\;\\ & \;\text{Relative Tonicity}\; & & \; \end{array}$$

(9) $$\begin{array}{llll} \text{EFWC} & ={\text{V}}_{\text{O}}*\left[1-\left({\text{O}}_{\left[\text{Na}+\text{K}\right]}∕{\text{P}}_{\left[\text{Na}+\text{K}\right]\text{pw}}\right)\right]\;\\ & \;\text{Relative Tonicity}\; & & \; \end{array}$$

**Figure 2:**
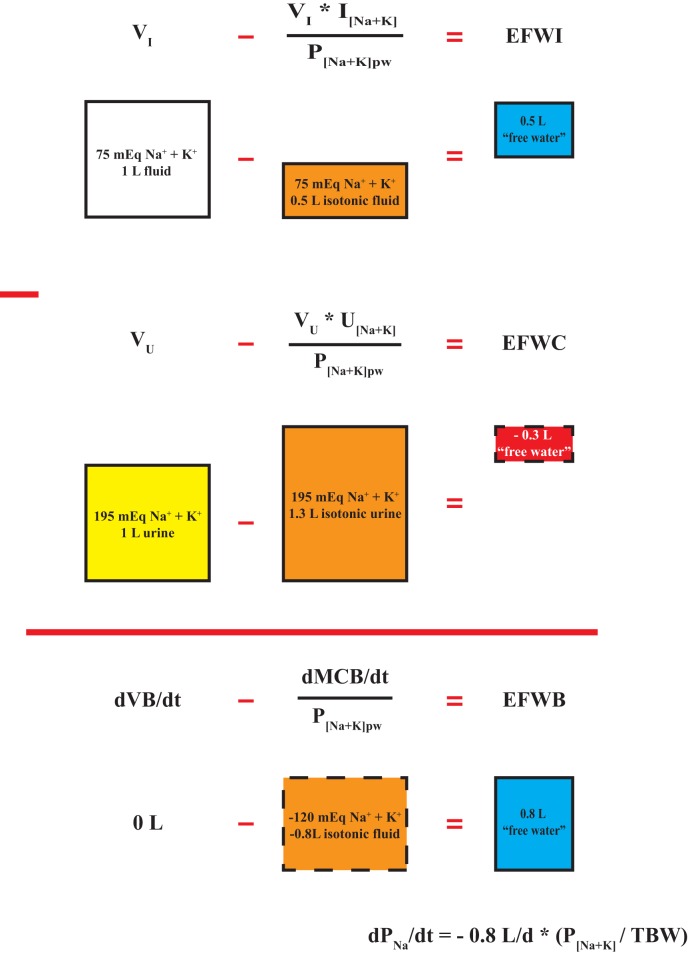
Segregation of inputs, outputs, and balances into isotonic and electrolyte-free water components. A hypothetical isotonic input rate is subtracted from total input volume rate to yield electrolyte-free water input (EFWI). Similarly, an isotonic urine output rate is subtracted from total urine flow rate to yield urinary EFWC. The difference between EFWI and electrolyte-free water clearance (EFWC) yields electrolyte-free water balance (EFWB), which is a composite of the rate of change in total fluid balance (dVB/dt) and monovalent cation balance (dMCB/dt). EFWB is proportional to dP_Na_/dt. Calculations are based on a plasma [Na + K]_pw_ equal to 150 mEq/L. The depicted boxes represent daily volumetric rates.

An alternative interpretation of EFWI and EFWC evaluates the difference between the rate of total input or output volume and an isotonic component:

(10) $$\begin{array}{llll} \text{EFWI} & ={\text{V}}_{\text{I}}-\left({\text{V}}_{\text{I}}*{\text{I}}_{\left[\text{Na}+\text{K}\right]}∕{\text{P}}_{\left[\text{Na}+\text{K}\right]\text{pw}}\right)\;\\ & \;\text{Total}\;\text{Isotonic}\; & & \; \end{array}$$

(11) $$\begin{array}{llll} \text{EFWC} & ={\text{V}}_{\text{O}}-\left({\text{V}}_{\text{O}}*{\text{O}}_{\left[\text{Na}+\text{K}\right]}∕{\text{P}}_{\left[\text{Na}+\text{K}\right]\text{pw}}\right)\;\\ & \;\text{Total}\;\text{Isotonic}\; & & \; \end{array}$$

where V_I_ and V_O_ are input and output flow rates and I_[Na+K]_ and O_[Na+K]_ are input and output Na + K concentrations, respectively. Eqs 10 and 11 are presented differently, but are mathematically equivalent to Eqs 8 and 9. The isotonic component term in Eqs 8 and 9 (in bold) is created by taking input or output [Na + K] expressed as mEq/L and multiplying by input or output flow rate (V_I_ or V_O_) in units of L per unit time to calculate monovalent cation input or output rate in mEq per unit time. The monovalent cation input or output rate (Na + K_i_ or Na + K_o_), expressed in mEq per unit time, is divided by plasma water [Na + K] in mEq/L (assuming water density equals 1) to create a volumetric rate expressed as L per unit time. This volumetric rate contains all of the Na^+^ and K^+^ input or output content with a [Na + K] equal to plasma water [Na + K] (i.e., isotonic). In the case of an isotonic input or output, the total volume in or out equals the isotonic component and EFWI or EFWC are zero. With hypotonic inputs or outputs, all of the Na^+^ and K^+^ content can be incorporated into an isotonic volume smaller than the total volume resulting in a positive EFWI or EFWC. Conversely, in the case of hypertonic inputs or outputs, all of the input or output Na^+^ and K^+^ content must be placed in an isotonic volume larger than total input or output volume leading to a negative EFWI or EFWC (Figure 2). Therefore, regardless of approach, negative EFWI and EFWC indicate hypertonic inputs and outputs, while positive EFWI and EFWC occur with hypotonic inputs and outputs.

EFWB is formed when EFWC is subtracted from EFWI:

(12) $$\begin{array}{ll} \frac{\begin{array}{cc} \text{EFWI} & ={\text{V}}_{\text{I}}-\left[{\left(\text{Na}+\text{K}\right)}_{\text{i}}∕{\text{P}}_{\left[\text{Na}+\text{K}\right]\text{pw}}\right]\\ -\text{EFWC} & ={\text{V}}_{\text{O}}-\left[{\left(\text{Na}+\text{K}\right)}_{\text{o}}∕{\text{P}}_{\left[\text{Na}+\text{K}\right]\text{pw}}\right] \end{array}}{\text{EFWB}=\left({\text{V}}_{\text{I}}-{\text{V}}_{\text{O}}\right)-\frac{{\left(\text{Na}+\text{K}\right)}_{\text{i}}-{\left(\text{Na}+\text{K}\right)}_{\text{o}}}{{\text{P}}_{\left[\text{Na}+\text{K}\right]\text{pw}}}} & \; \end{array}$$

where V_I_ and V_O_ are input and output flow rates (volume per unit time) and (Na + K)_i_ and (Na + K)_o_ are Na + K input and output rates in mEq per unit time (Figure 2). To simplify the expression, we set V_I_–V_O_ = dVB/dt (rate of change in volume balance) and (Na + K)_i_–(Na + K)_o_ = dMCB/dt (rate of change in monovalent cation balance) to yield a short hand form of EFWB (Figure 2):

(13) $$\text{EFWB}=\text{dVB}∕\text{dt}-\frac{\text{dMCB}∕\text{dt}}{{\text{P}}_{\left[\text{Na}+\text{K}\right]\text{pw}}}$$

If the rate of change in total fluid balance (dVB/dt) is completely accounted for by a change in isotonic volume (dMCB/dt ÷ P_[Na+K]pw_), then EFWB is zero and plasma [Na^+^] does not change. Otherwise, if total fluid balance exceeds isotonic fluid, EFWB is positive, and plasma [Na^+^] falls and *vice versa*.

Taken together, EFWB is *not* simply fluid or volume balance (i.e., fluid volume in minus volume out) added or subtracted from TBW as often interpreted (37, 46), but is a composite volumetric rate reflecting rates of change in Na^+^, K^+^, and water balance. Based on the relationship between dP_Na_/dt and EFWB, dysnatremias are disorders of persistently positive or negative EFWB, which involve fluid and monovalent cation balance derangements rather than simply “too much” or “too little” water. H OMEOSTASIS]

## EFWB in Daily Maintenance of Normal Plasma [Na^+^] Homeostasis

To understand the pathogenesis of dysnatremias, we first examine how steady state plasma [Na^+^] is maintained. In the normal physiologic state over the long term, EFWB is actively regulated to zero so that dP_Na_/dt equals zero and plasma [Na^+^] remains stable around a homeostatic set point. The genesis of dysnatremias, therefore, requires a period of positive EFWB for hypotonic hyponatremia or negative EFWB for hypernatremia. A chronic steady state of altered plasma [Na^+^] may occur with EFWB returning to zero, but represents an “inappropriately zero” EFWB in the setting of dysnatremia. Rather, EFWB should be negative in hypotonic hyponatremia and positive in hypernatremia to normalize plasma [Na^+^]. It is thus useful to see how the body normally utilizes the concept of EFWB in health to prevent dysnatremias.

Although EFWI and EFWC have thus far been considered as a single input or output respectively, EFWI and EFWC can actually be calculated for each input and output and simply added together to yield an EFWB for multiple inputs and outputs as it occurs in daily life. We first consider EFWI for “unmeasured” inputs and then follow with a discussion on EFWC in terms of “unmeasured” outputs. Unmeasured inputs consist of food water content and metabolically produced water from fuel oxidation, while unmeasured outputs include insensible losses, sweat, and stool.

In terms of unmeasured inputs, food water content is often ignored, but contributes significantly to water balance. Depending on diet composition, about 20–25% of oral water or 0.5–0.7 L per day comes from solid food rather than beverages (47, 48). Metabolic water production is typically estimated at about 0.3 L per day yielding a total, unmeasured water input of 0.8–1 L per day (47).

Insensible losses, named for their inability to be perceived, represent water evaporation through the skin and expired air at an estimated rate of about 420 mL per m^2^ body surface area (or 10 mL/kg in relatively normal sized individuals) split in about a 60–40 ratio between skin (~250 mL per m^2^) and lungs (~170 mL per m^2^) in a normal person. Small variations do occur with ambient and body temperature and tidal volume, but the only clinically significant change occurs with mechanical ventilation. Since most ventilators use humidified air, respiratory insensible losses are cut by about 80% in this situation (49).

Sweat, though unmeasured, is a perceptible fluid with electrolyte content and should not be confused with pure water, insensible losses. Sweat [Na^+^] varies widely from about 15–75 mEq/L in direct proportion to sweating rate (range about 0.1–1 L/h), while sweat [K^+^] is relatively minor and constant between 2.5 and 6.5 mEq/L (50–52). In the hospitalized setting, fever is the main cause of sweating, yet very little is known about sweat rates and ionic composition in febrile patients. In one study, patients demonstrated observable sweating when rectal temperatures exceeded 39.5^o^C (103.1^o^F) with rates of about 0.1–0.15 L/h leading to about 0.6 L of sweat loss per day as patients were febrile for about 4–6 h (53). At these sweat rates, one could estimate a sweat [Na + K] of 20–30 mEq/L.

Patients consuming a regular diet with normal bowel function have stool water losses of about 100–150 mL per day (normal daily stool output <200 g/day with 75% water). Normal stool [Na + K] is typically near 140 mEq/L and essentially isotonic to plasma (54, 55). Classically, secretory diarrheas exhibit little change in stool [Na + K], while osmotic diarrheas demonstrate lower stool [Na + K] in the range of 25–100 mEq/L (55, 56).

Based on our discussion, we can calculate a near zero EFWB for unmeasured fluid fluxes in a normal, sedentary patient (Table 1). Thus, in most people, urinary EFWC matches oral beverage and electrolyte intake EFWI to maintain neutral EFWB and plasma [Na^+^]. The high correlation between oral beverage intake and urine output in healthy adults corroborates this notion (47, 49, 57). On a typical American diet, mean urine volume is about 1.5 L, urinary Na^+^ excretion 150 mEq/d, and urinary K^+^ excretion 50 mEq/d (assuming normal P_[Na+K]_ = 144 mEq/L), which produces a typical urinary EFWC near zero (about +0.2 L) and equal to oral EFWI (58). Thirst primarily regulates oral EFWI, while ADH modulates urine concentration to alter urinary EFWC. Both thirst and ADH action work in concert to target EFWB to zero and maintain plasma [Na^+^]. Equally important, not only is overall EFWB regulated to zero in the long term, but so are its components, the rate of change in volume balance and monovalent cation balance, as these must also be in steady state to maintain body fluid compartments and their composition within narrow physiological limits. For instance, most edematous patients are normonatremic with positive volume and cation balances that yield an EFWB = 0 and no change in plasma [Na^+^] (59, 60). Similarly, patients with cholera may rapidly lose liters of isotonic fluid with little change in EFWB and plasma [Na^+^] (61). Thus, normal body fluid homeostasis requires regulation of EFWB to zero, and its components, namely Na^+^, K^+^, and fluid balance.

**Table 1:** “Unmeasured” electrolyte-free water input (EFWI) and electrolyte-free water clearance (EFWC) in a normal sedentary patient.

	V_I_ (per day)	I_[Na+K]_	EFWI (per day)
Metabolic water	0.3 L	0	0.3 L
Food water content	0.5–0.7 L	0	0.5–0.7 L

Total			0.8–1.0 L

	**V_O_ (per day)**	**O_[Na+K]_**	**EFWC (per day)**

Insensible losses	0.7–0.9 L	0	0.7–0.9 L
Sweat	0	25 mEq/L	0
Stool	0.1–0.15 L	≅P_[Na+K]_	0

Total			0.7–0.9 L

## Application of the EFWB Concept for the Treatment of Dysnatremias

The classic approach to treatment of dysnatremias involves the calculation of a monovalent cation deficit (also known as sodium deficit) or TBW excess for hyponatremia and TBW deficit for hypernatremia (62). The monovalent cation deficit calculation presumes that the entire desired rise in plasma [Na^+^] will occur with positive cation balance while TBW remains constant. Unfortunately, most cases of hyponatremia do not involve a pure loss of cation to justify this therapeutic strategy (29–32, 63–65). TBW excess and deficit calculations lie at the other end of the spectrum and presume that changes in TBW will account for the entire desired change in plasma [Na^+^] with no alteration in monovalent cation balance. These calculations are often termed “free water” excess or deficits, but this is inherently misleading as it equates total volume balance (dVB/dt) with free water balance (EFWB) without accounting for monocation balance.

So how can we integrate monocation and volume balances in the treatment of dysnatremias? We suggest that ECF volume status and repair along with EFWB should be considered. Rearrangement of Eq. 10 shows that the total volumetric rate of fluid administration to a given individual (dVB/dt) can be conceptualized as having two distinct components: a pure water component (EFWB) as well as a isotonic component (dMCB/dt ÷ P_[Na+K]pw_). Thus, if one can define EFWB and a rate of isotonic repletion (dMCB/dt ÷ P_[Na+K]pw_), then a total rate of volume administration that will account for both free water and cation balance simultaneously can be determined. Thus, it becomes imperative to be able to concretely define EFWB and dMCB/dt.

A target EFWB required to achieve a desired rate of plasma [Na^+^] correction (R), can be delineated by replacing dP_Na_/dt with a rate of correction (R) in the EFWB-dP_Na_/dt relationship in Eq. 3:

(14) $$\text{EFWB}=-\text{R}*\text{TBW}∕{\text{P}}_{\left[\text{Na}+\text{K}\right]}$$

For example, if we wished for plasma [Na^+^] to change by 6 meq/L/day, R would equal 6 and one could define EFWB explicitly using Eq. 14 and an estimation of TBW. Admittedly, the estimation of TBW is far from perfect. TBW is a function of body composition and results from a weighted average of tissue water content. Most tissues, such as visceral organs and skin, consist of 70–80% water by weight, while bone and adipose tissue are only 10–20% water. Thus, subjects with relatively low muscle mass or increased adiposity will have a lower TBW relative to body weight. The elderly with preferential loss of muscle mass with age and women with higher body fat content have diminished TBW relative to weight. Absolute TBW increases with obesity, but decreases relative to weight with the gain of “dry” adipose tissue, while the opposite occurs in cachectic individuals. We classically estimate TBW at the bedside as 60% of weight in kilograms for men, 50% for women, and 5% less from these values in elderly adults (2). Improved TBW estimates for relatively healthy individuals may be obtained using equations based on anthropometric variables (66, 67). However, dysnatremic, hospitalized patients pose a greater challenge to TBW estimation. Besides the alterations in body composition that occur in disease to modify TBW, the pathophysiology of most dysnatremias involves an element of altered TBW, which is difficult to quantify at the time of clinical presentation. Euvolemic and hypervolemic hyponatremic patients have higher than predicted TBW, while dehydrated, hypernatremic patients exhibit lower TBW. As a hypothetical example, a young man with recent onset euvolemic or hypervolemic hyponatremia may present with a body weight of 70 kg, but his unknown pre-morbid baseline weight is 65 kg. Therefore, we may be relatively accurate in estimating his pre-morbid TBW as 39 L (0.6*65 kg), but if we utilize the same rule of thumb with his presentation weight of 70 kg, we would calculate a presenting TBW of 42 L (0.6*70 kg), instead of the “true” presentation TBW of 44 L (pre-morbid TBW + presumed fluid weight gain of 5 kg). While a careful assessment of body weight trends is useful in improving our estimates, it is easy to imagine that accounting for complex body compositional changes is not straightforward at the bedside. However, all approaches to design therapy for dysnatremias depend on an estimation of TBW. In the absence of reliable body weight trends, we utilize presentation body weight with minor adjustments for alterations in body composition (e.g., obesity, cachexia) and the dysnatremia at hand.

Defining dMCB/dt *a priori* is also difficult, but can be made easier by integrating it with the concept of ECF volume regulation. Changes in ECF volume often occur concurrently with dysnatremias, and thus provide a good starting point to be able to integrate qualitative physiology as often taught in textbooks with quantitative modeling. In general, isotonic sodium gain or pure water gain may expand ECF. 1 L of pure water expands TBW by 1 L and the ECF by the fraction of TBW composed of ECF (f_ECF_). f_ECF_ is normally about 0.4–0.45 or 40–45% of TBW (2, 20), though one can estimate lower fractions with ECF depletion or ICF expansion such as 0.33. Conversely, 1 L of isotonic sodium expands the ECF by the entire volume of 1 L (3, 62). Based on these concepts, one can then write the following relationship for the rate of change in ECF volume (dECFV/dt):

(15) $$\begin{array}{l} \frac{\text{dECFV}}{\text{dt}}=\frac{\text{dNaB}∕\text{dt}}{{\text{P}}_{\left[\text{Na}+\text{K}\right]\text{pw}}}+\left({\text{f}}_{\text{ECF}}*\text{ EFWB}\right)\\ \text{Isotonic}\;\text{“Pure Water”} \end{array}$$

or

(16) $$\text{dNaB}∕\text{dt}={\text{P}}_{\left[\text{Na}+\text{K}\right]\text{pw}}*\left[\text{dECFV}∕\text{dt}-\left({\text{f}}_{\text{ECF}}*\text{EFWB}\right)\right]$$

In assessing a patient, one usually estimates the degree of ECF volume contraction or expansion and thus by inference, also assumes a given volume goal for that patient when trying to correct ECF volume. From the above equation, if one sets dECFV/dt to a certain value (i.e., 1 L/24 h) and has already defined EFWB using Eq. 14, then dNaB/dt can be determined. The term dMCB/dt is composed of the composite change in sodium and potassium balance per unit time (dNaB/dt and dKB/dt). If there is a certain degree of hypokalemia involved, one can empirically choose a K replacement strategy (i.e., 40 meq/24 h) as a surrogate for dKB/dt. It should be noted that disorders of potassium are inherently complex, with plasma K values not only being determined by intake and losses, but also, in some cases, shifts between the extracellular and intracellular fluid spaces. That being said, at this time there are no concrete mathematical models to accurately determine K deficit. Thus empiric K repletion, while not ideal, represents a practical first step to more accurately design a therapeutic regimen at the bedside. With this in mind the calculation of dNaB/dt and the addition of the empirically chosen K strategy allow quick calculation of dMCB/dt and by proxy the amount of volume administration that will correct cation and EFWBs that caused the dysnatremia.

Thus, a stepwise method for arriving at a treatment plan for a given dysnatremia can now be defined based on the relationships defined above:

1. Define an EFWB target rate by using Eq. 14 and a desired rate of plasma [Na^+^] correction, current plasma [Na^+^] concentration, and an estimate of TBW.
2. Based on clinical assessment, estimate the degree of ECF volume alteration desired over a given period of time (dECFV/dt). Use the estimate of ECF volume change and EFWB to define the rate of change in sodium content (dNaB/dt).
3. Determine if any K deficit is present that needs to be empirically corrected and add it to dNaB/dt to determine dMCB/dt, the composite rate of change in cation mass balance.
4. Use Eq. 13 to calculate the total required change in volume administered (dVB/dt) to correct the plasma [Na^+^] at the desired rate. This involves adding EFWB that was calculated in step 1 to dMCB/dt÷P_[Na+K]pw_ calculated in step 4.
5. Define an infusion or oral therapy that provides the calculated Na^+^, K^+^, and volume goals.

We illustrate the application of this process with two illustrative cases of hyponatremia. A case of hypernatremia is provided in the Data Sheet S1 in Supplementary Material.

### Case 1: SIADH Secondary to Pneumonia

A 68-year-old gentleman was admitted with fever, productive cough, and left-sided pleuritic chest pain and diagnosed with left lower lobe pneumonia accompanied by a complicated parapneumonic effusion. A pigtail catheter was placed for effusion drainage, which further required intrapleural DNase and tPA to facilitate clearance. Of note, the patient also attested to a 30 pound weight loss over the previous year and notably had a remote history of gastrointestinal stromal tumor in remission after medical therapy. Physical examination at the time of consultation was unremarkable except for cachexia and a weight of 52 kg (body mass index of 16.4 kg/m^2^). Over the course of the 10-day hospitalization, plasma [Na^+^] had declined from 137 to 128 mEq/L despite a 1–1.5 L fluid restriction. Plasma chemistries were also notable for a [K^+^] of 4 mEq/L, BUN of 8 mg/dL, and a creatinine of 0.4 mg/dL. Thyroid studies and ACTH stimulation test were normal. Urine chemistries were as follows: [Na^+^] = 75 mEq/L, [K^+^] = 64 mEq/L, osmolality = 546 mOsm/kg, urine urea nitrogen 644 mg/dL, urine creatinine 86 mg/dL. Urine output was about 800 mL/day.

To use the stepwise approach, we estimated TBW at 31.2 L (0.6*52 kg) using 60% as the percent water weight to reflect the increase in TBW relative to weight due to cachexia and SIADH-associated overhydration in spite of the patient’s advanced age. f_ECF_ was estimated at 0.4 which is near normal, reflecting a balance between cachexia with reduced muscle mass and ICF, and SIADH hyponatremia which tends to expand ICF more than ECF. With P_[Na+K]_ = 132 mEq/L and P_[Na+K]pw_ = 142 (assumes normal plasma water fraction of 93%), a modest target for rise in plasma [Na^+^] of 3 mEq/L/day was chosen.

#### Step 1: Define EFWB Target Rate

$$\begin{array}{llll} & \text{EFWB}=-\text{R}*\text{TBW}∕{\text{P}}_{\left[\text{Na}+\text{K}\right]}\; & & \;\\ & \text{EFWB}=-\text{3 mEq}∕\text{L}∕\text{day}*31.2\text{ L}∕\text{132 mEq}∕\text{L}\; & & \;\\ & \;\;\;=-0.71\text{ L}∕\text{day}\; & & \; \end{array}$$

#### Step 2: Define Na^+^ Balance Rate Using ECF Volume and EFWB Target Rates

$$\begin{array}{llll} & \text{dECFV}∕\text{dt}=0\left(\text{maintain euvolemia}\right)\; & & \;\\ & \text{dNaB}∕\text{dt }={\text{P}}_{\left[\text{Na}+\text{K}\right]\text{pw}}*\left[\text{dECFV}∕\text{dt }-\left({\text{f}}_{\text{ECF}}*\text{EFWB}\right)\right]\; & & \;\\ & \text{dNaB}∕\text{dt }=\text{142 mEq}∕\text{kg}*[0-\left(0.4*-0.71\text{ L}∕\text{day}\right)\; & & \;\\ & \;\;\;\;=40\text{ mEq}∕\text{day}\; & & \;\\ & \text{dNaB}∕\text{dt}=\text{Input N}{\text{a}}^{+}\text{Rate}-\text{Urinary N}{\text{a}}^{+}\text{ Excretion Rate}\; & & \; \end{array}$$

$$\begin{array}{llll} & \text{Given that the urinary N}{\text{a}}^{+}\text{Excretion Rate}\; & & \;\\ & \;=\text{75 mEq}∕\text{L}*0.8\text{ L}∕\text{day}=60\text{mEq}∕\text{day}\; & & \;\\ & \text{Input N}{\text{a}}^{+}\text{Rate}=\text{dNaB/dt}+\text{Urinary N}{\text{a}}^{+}\text{Excretion Rate}\; & & \;\\ & \;\;\;\;=40+60=100\text{mEq/day}\; & & \; \end{array}$$

#### Step 3: Determine if any K^+^ Supplementation Is Required and Isotonic Volume Rate

$$\begin{array}{llll} \text{dKB}∕\text{dt} & =0\;\left(\text{no }{\text{K}}^{+}\text{supplementation planned}\right)\; & & \;\\ \frac{\text{dMCB}∕\text{dt}}{{\text{P}}_{\left[\text{Na}+\text{K}\right]\text{pw}}} & =\frac{\text{dNaB}∕\text{dt}}{{\text{P}}_{\left[\text{Na}+\text{K}\right]\text{pw}}}+\frac{\text{dKB}∕\text{dt}}{{\text{P}}_{\left[\text{Na}+\text{K}\right]\text{pw}}}\; & & \;\\ \frac{\text{dMCB}∕\text{dt}}{{\text{P}}_{\left[\text{Na}+\text{K}\right]\text{pw}}} & =\frac{40}{142}+\frac{0}{142}=0.28\text{ L}∕\text{day}\; & & \; \end{array}$$

#### Step 4: Calculate Volume Balance Rate Based on EFWB Target and Isotonic Volume Rates

$$\text{dVB}∕\text{dt}=\text{EFWB}+\frac{\text{dMCB}∕\text{dt}}{{\text{P}}_{\left[\text{Na}+\text{K}\right]\text{pw}}}=-0.71+0.28=-0.43\text{ L}∕\text{day}$$

$$\text{dVB}∕\text{dt}={\text{V}}_{\text{IVF}}+{\text{V}}_{\text{oral}}+{\text{V}}_{\text{unmeasured}}-{\text{V}}_{\text{urine}}=-0.43\text{ L}∕\text{day}$$

The patient was eating about half his diet so we assumed insensible losses exceeded food water content by 0.25 L/day. He was also receiving 150 mL of IV piperacillin–tazobactam dissolved in NS. Based on this, one could use the following estimates:

$$\begin{array}{llll} {\text{V}}_{\text{unmeasured}} & =-0.25\text{ L}\; & & \;\\ {\text{V}}_{\text{IVF}} & =0.15\text{ L}∕\text{day}\; & & \;\\ {\text{V}}_{\text{urine}} & =0.8\text{ L}∕\text{day}\; & & \;\\ {\text{V}}_{\text{oral}} & =\text{dVB/dt}-{\text{V}}_{\text{IVF}}-{\text{V}}_{\text{unmeasured}}+{\text{V}}_{\text{urine}}\; & & \;\\ & =-0.43-0.15+0.25+0.8=047\text{ L/day}\; & & \; \end{array}$$

In light of these calculations, the patient was placed on a fluid restriction of two 8 ounce cups a day (about 480 mL) and prescribed 1 g po bid of NaCl (~34 mEq Na^+^) to provide the approximate Na^+^ for the required positive balance above and beyond what was already being provided by dietary intake and antibiotic-associated IV fluids (assumed from the pre-treatment urinary Na^+^ excretion rate).

To compare strategies, we next designed a therapeutic regimen utilizing a classic approach based on TBW excess. The TBW excess associated with the desired 3 mEq/L change in plasma [Na^+^] from 128 to 131 mEq/L is calculated as follows:

$$\begin{array}{llll} & \text{TBW Excess}=\text{TBW}*[(\text{Plasma}{\left[{\text{Na}}^{+}\right]}_{\text{f}}∕\text{Plasma}{\left[{\text{Na}}^{+}\right]}_{\text{i}})-1)]\; & & \;\\ & \text{TBW Excess}=31.2\text{ L}*\left[\left(131∕128\right)-1\right]=0.73\text{ L}\; & & \; \end{array}$$

Thus, to achieve the desired change in plasma [Na^+^] from 128 to 131 mEq/L, we would plan on a negative fluid balance of 0.73 L to target the calculated TBW excess:

$$\begin{array}{llll} \text{Desired Fluid Balance} & ={\text{V}}_{\text{IVF}}+{\text{V}}_{\text{oral}}+{\text{V}}_{\text{unmeasured}}-{\text{V}}_{\text{urine}}\; & & \;\\ & =-0.73\text{ L}∕\text{day}\; & & \; \end{array}$$

$$\begin{array}{llll} {\text{V}}_{\text{unmeasured}} & =-0.25\text{ L}\; & & \;\\ {\text{V}}_{\text{IVF}} & =0.15\text{ L}∕\text{day}\; & & \;\\ {\text{V}}_{\text{urine}} & =0.8\text{ L}∕\text{day}\; & & \;\\ {\text{V}}_{\text{oral}} & =\text{Fluid Balance}-{\text{V}}_{\text{IVF}}-{\text{V}}_{\text{unmeasured}}+{\text{V}}_{\text{urine}}\; & & \;\\ & =-0.73-0.15+0.25+0.8=0.17\text{ L/day}\; & & \; \end{array}$$

The TBW excess approach calculates a fluid restriction of 170 mL/day, even more onerous than the fluid restriction prescribed by our proposed approach. The reason for the difference is that the TBW excess approach assumes that cation balance (dMCB/dt) is equal to zero. That is, volume balance accounts wholly for EFWB. In fact, positive Na^+^ balance during the correction of SIADH-associated hyponatremia with fluid restriction is well described and may contribute to the rise in plasma [Na^+^] (31, 68–70). In essence, negative fluid balance created with fluid restriction leads to clinically unnoticed ECF volume depletion engendering positive Na^+^ balance for correction. Thus, in our case, a loss of 0.71 L/day of EFW raises plasma [Na^+^] by 3 mEq/L/day, but also depletes the ECF by 0.28 L/day (0.4*0.71 L/day). This ECFV deficit is “added back” to restore ECF volume as isotonic NaCl, which does not affect plasma [Na^+^].

In this case, as in many cases, the two 8 ounce cups per day beverage restriction proved to be difficult. The plasma [Na^+^] the following day was unchanged at 128 mEq/L and the patient admitted to drinking 4 or more cups of fluid. Given the challenging fluid restriction, we wanted to examine additional therapies to liberalize the fluid restriction in this case. NaCl supplementation is often used as an adjunct to fluid restriction for the treatment of SIADH. But how does NaCl supplementation affect EFWB? Since total solute excretion rate and urine osmolality determine urine flow rate (71), we can alternatively express urinary EFWC as:

(17) $$\text{Urinary EFWC}=\frac{\text{OE}{\text{R}}_{\text{urine}}}{{\text{U}}_{\left[\text{osm}\right]}}-\frac{{\left(\text{Na}+\text{K}\right)}_{\text{urine}}}{{\text{P}}_{\left[\text{Na}+\text{K}\right]\text{pw}}}$$

where OER_urine_ is the total urinary osmole excretion rate and (Na + K)_urine_ is the daily urinary monovalent cation excretion rate (equals V_u_*U_[Na+K]_). The change in urinary EFWC with NaCl supplementation can be approximated as:

(18) $$\Delta \text{ Urinary EFW}{\text{C}}_{\text{salt}}=\frac{2*\text{N}{{\text{a}}^{+}}_{\text{sup}}}{{\text{U}}_{\left[\text{osm}\right]}}-\frac{2*\text{N}{{\text{a}}^{+}}_{\text{supp}}}{2*{\text{P}}_{\left[\text{Na}+\text{K}\right]\text{pw}}}$$

where Na^+^_supp_ is the amount of NaCl supplementation provided in mEq Na^+^ per day. Thus, when U_[osm]_ is greater than 2*P_[Na+K]pw_ as in this case (546 mOsm/kg vs. 284 mEq/L), increasing the osmolar excretion rate (OER_urine_) through NaCl supplementation paradoxically *decreases* urinary EFWC, an effect which would tend to increase EFWB and worsen hyponatremia. So why does salt supplementation work in many cases of SIADH? Luckily, pure NaCl ingestion decreases EFWI more than the fall in EFWC resulting in a net overall decrease in EFWB and rise in plasma [Na^+^]. Since humans cannot urinate salt crystals, a pure NaCl load *sans* water obligates some degree of urinary volume loss which underlies the rise in plasma [Na^+^]. Thus, in our patient, if we wanted to increase the fluid restriction to 1 L/day, we would need to increase urine volume by about 0.5 L/day requiring an extra 273 mosm (546 mOsm/kg*0.5 L) of solute or 136.5 mEq NaCl per day. If we add to this value the requisite 40 mEq/day positive Na^+^ balance, we must provide 176.5 mEq of NaCl per day (about ten 1 g NaCl tablets).

Disadvantages of NaCl supplementation include a potential for excessive ECF volume expansion or increased thirst which may counteract compliance with the relaxed fluid restriction. Thus, another approach is to utilize loop diuretics to block urinary concentration and increase urinary EFWC (72). Previous reports utilize 40 mg oral furosemide once daily to create a daily urine volume of about 1.5–2 L per day. In the case of SIADH, where we want to maintain euvolemia, high Na^+^ intake and the post-diuretic, rebound Na^+^ retention is leveraged to create a slightly positive Na^+^ balance (73), which we assume to be the previously calculated requirement of 40 mEq/day. While the exact amount of Na^+^ intake required to accomplish this is uncertain, a daily Na^+^ intake of about 150 mEq is reasonable and would require about 90 mEq of supplemental Na^+^ (five to six 1 g NaCl tablets) assuming the patient’s pre-treatment intake is in balance with urinary excretion at about 60 mEq/day. Thus, in our patient, assuming a diuretic induced daily urine output of 1.5 L, we can now liberalize the fluid intake to 1.2 L per day, since urine output has now increased by 0.7 L.

Urea supplementation is an alternative to liberalize fluid intake in the treatment of SIADH (74). In contrast to NaCl supplementation, addition of urea increases non-electrolyte excretion rate, and thus effectively raises urinary EFWC to counterbalance the increase in oral EFWI due to liberalization of the fluid restriction. Thus, adding 273 mosm of urea (about 16 g urea) increases urinary volume by 0.5 L (273 ÷ 546 mOsm/kg) and allows us to liberalize the fluid restriction to 1 L.

### Case 2: Thiazide Diuretic-Associated Hyponatremia

Thiazide diuretic-associated hyponatremia is often multifactorial involving a combination of diuretic-induced hypovolemia promoting ADH release, blockade of distal convoluted tubule urinary dilution, and polydipsia (75). We present a case of a 76-year-old Asian woman with a medical history notable for hypertension treated with ACE inhibitor and thiazide diuretic who initially presented to the hospital with nausea and RLQ abdominal pain diagnosed as cecal diverticulitis by CT imaging. Anti-hypertensives were held and the patient was treated conservatively with bowel rest, IV fluids, and antibiotics (ciprofloxacin and metronidazole). She defervesced over a course of 3 days and was discharged tolerating a full liquid diet. Notably, her blood pressures became elevated on hospital day 2 and she was restarted on her previous home anti-hypertensive regimen, which included 50 mg hydrochlorothiazide daily. The patient presented for follow-up with her PCP 5 days after discharge with minimal abdominal pain, but stating that her food intake was limited mainly to small amounts of rice due to nausea and bloating. She denied vomiting or altered bowel pattern. She had noted diminished urine output and drank as much water as possible to “help her kidneys.” While she was quite fatigued with generalized weakness, her mental status was completely unchanged according to her PCP and family. She denied headache or focal neurologic symptoms. Physical examination was notable for supine BP of 167/74, HR 64, standing BP of 133/65, HR 80, weight 44 kg, mucous membranes mildly dry, JVP visible only at <30^o^, and no edema. Laboratory studies on admission were notable for a plasma [Na^+^] = 106 mEq/L, plasma [K^+^] = 3.2 mEq/L, urine [Na^+^] = 33 mEq/L, urine [K^+^] = 21 mEq/L, and urine osmolality = 535 mOsm/kg H_2_O. Repeat CT abdomen demonstrated improved cecal thickening and stranding. Bladder catheterization yielded a urine flow rate of about 20 mL/h.

Based on the presentation of hypotonicity and clinical features of volume depletion, we hypothesized that the patient’s ICF was expanded with a depleted ECF. Na_e_ was significantly depressed and K_e_ mildly diminished. Thus, the goals of therapy were to replete ECF and slowly raise serum plasma [Na^+^] and provide K^+^ supplementation. Based on her age, gender, and hydration status in hyponatremia, we estimated TBW at 22 L (44 kg*0.5). Given the ICF expansion and ECF depletion, f_ECF_ was estimated at 0.33. For the first 24 h, we targeted an increase in plasma [Na^+^] of 6 mEq/L/day, ECFV gain of 1 L, and positive K^+^ balance of 40 mEq and applied the stepwise approach.

#### Step 1: Define EFWB Target Rate

$$\begin{array}{llll} & \text{EFWB}=-\text{R}*\text{TBW}∕{\text{P}}_{\left[\text{Na}+\text{K}\right]}\; & & \;\\ & \text{EFWB}=-\text{6 mEq}∕\text{L}∕\text{day}*22∕10\text{9 mEq}∕\text{L}=-1.2\text{ L}∕\text{day}\; & & \; \end{array}$$

#### Step 2: Define Na^+^ Balance Rate Using ECF Volume and EFWB Target Rates

$$\begin{array}{llll} & \text{dECFV}∕\text{dt}=+1\text{ L}∕\text{day}\; & & \;\\ & \text{dNaB}∕\text{dt}={\text{P}}_{\left[\text{Na}+\text{K}\right]\text{pw}}*\left[\text{dECFV}∕\text{dt}-\left({\text{f}}_{\text{ECF}}*\text{EFWB}\right)\right]\; & & \;\\ & \text{dNaB}∕\text{dt}=\text{117 mEq}∕\text{L}*[1-\left(0.33*1.2\text{ L}\right)=\text{164 mEq}∕\text{day}\; & & \;\\ & \text{dNaB}∕\text{dt}=\text{IV N}{\text{a}}^{+}\text{Infusion Rate}-\text{Urinary N}{\text{a}}^{+}\text{Excretion}\; & & \;\\ & \text{Urinary N}{\text{a}}^{+}\text{Excretion}=\text{33 mEq}∕\text{L}*0.48\text{L}∕\text{day}\; & & \;\\ & \;=15.\text{8 mEq}∕\text{day}\; & & \;\\ & \text{N}{\text{a}}^{+}\text{Infusion Rate}=\text{dNaB}∕\text{dt}+\text{Urinary N}{\text{a}}^{\text{+}}\text{Excretion}\; & & \;\\ & \;=164+15.8=179\text{ mEq/day}\; & & \; \end{array}$$

#### Step 3: Determine if Any K^+^ Supplementation Is Required and Isotonic Volume Rate

$$\begin{array}{llll} & \text{dKB}∕\text{dt}=+40\text{ mEq}∕\text{day}\; & & \;\\ & \text{dKB}∕\text{dt}=\text{IV}{\text{K}}^{+}\text{Infusion Rate}-\text{Urinary }{\text{K}}^{+}\text{Excretion Rate}\; & & \;\\ & \text{IV}{\text{K}}^{+}\text{Infusion Rate}=\text{dKB/dt}+\text{Urinary }{\text{K}}^{+}\text{Excretion Rate}\; & & \;\\ & \;=40+10=50\text{ mEq/day}\; & & \;\\ & \frac{\text{dMCB}∕\text{dt}}{{\text{P}}_{\left[\text{Na}+\text{K}\right]\text{pw}}}=\frac{\text{dNaB}∕\text{dt}}{{\text{P}}_{\left[\text{Na}+\text{K}\right]\text{pw}}}+\frac{\text{dKB}∕\text{dt}}{{\text{P}}_{\left[\text{Na}+\text{K}\right]\text{pw}}}\; & & \;\\ & \frac{\text{dMCB}∕\text{dt}}{{\text{P}}_{\left[\text{Na}+\text{K}\right]\text{pw}}}=\frac{164}{117}+\frac{40}{117}=1.74\text{ L}∕\text{day}\; & & \; \end{array}$$

#### Step 4: Calculate Volume Balance Rate Based on EFWB Target and Isotonic Volume Rates

$$\text{dVB}∕\text{dt}=\text{EFWB}+\frac{\text{dMCB}∕\text{dt}}{{\text{P}}_{\left[\text{Na}+\text{K}\right]\text{pw}}}=-1.2+1.74=0.54\text{ L}∕\text{day}$$

$$\begin{array}{llll} & \text{dVB}∕\text{dt}={\text{V}}_{\text{IVF}}+{\text{V}}_{\text{oral}}+{\text{V}}_{\text{unmeasured}}-{\text{V}}_{\text{urine}}=0.54\text{ L}∕\text{day}\; & & \;\\ & \text{Assuming}{\text{V}}_{\text{oral}}+{\text{V}}_{\text{unmeasured}}\; & & \;\\ & \;=0\left(\text{fluid intake offsets insensible losses}\right),\; & & \;\\ & {\text{V}}_{\text{IVF}}=\text{dVB}∕\text{dt}+{\text{V}}_{\text{urine}}=0.54\text{ L/day}+0.48\text{ L/day}\; & & \;\\ & \;=1.02\text{ L/day}\; & & \; \end{array}$$

We allowed the patient to drink up to 750 mL/day of beverages (V_oral_) and assumed that this offset the fluid loss from unmeasured fluid balance (V_unmeasured_), since the patient was not eating food. Thus, we needed to provide 179 mEq NaCl and 50 mEq KCl in 1.02 L of IVF. To make this clinically straightforward, we prescribed NS + 40 mEq KCl at 45 mL/h for 24 h to deliver 1.08 L fluid, 166 mEq NaCl, and 44 mEq KCl. Plasma [Na^+^] was measured every 6 h and after 24 h, plasma [Na^+^] had risen to 112 mEq/L.

Taken together, we have designed therapeutic regimens to treat two complex hyponatremia cases using the concept of EFWB and ECF volume repair. In doing so, we have highlighted its advantages including clear identification of goals for plasma [Na^+^] correction rate, EFWB, and ECF volume; a concrete step-by-step procedure to achieve these targets; and a simple way to rationalize therapy associated alterations in body fluid compartments.

## Review of the Quantitative Approaches to Dysnatremias

Many previous studies, including the approach described herein, have promulgated approaches to dysnatremias using mathematical manipulations of the idealized or empiric Edelman plasma [Na^+^] relationship (37, 39, 40, 46, 76). In the introduction, we discussed the shortcomings of these relationships and thus all of the described approaches are limited by the assumptions of the underlying plasma [Na^+^] relationship. Nguyen and colleagues have suggested that the empiric Edelman equation may be more accurate as its “non-ideal” slope and *y*-intercept accounts for tonicity disequilibrium between body fluid compartments and Na^+^ “storage” in connective tissues though these non-ideal values are assumed to be constant. Any approach using the idealized plasma [Na^+^] relationship may be converted to a more complex form using the empiric Edelman equation as done by Nguyen and colleagues (39).

All of the quantitative approaches to dysnatremias are based on the idea of keeping a balance sheet of monocation mass balance alongside fluid balance and then using these balances with the chosen plasma [Na^+^] relationship. Thus, the approaches depend on an accurate accounting of inputs and outputs, including unmeasured inputs such as metabolic water production and unmeasured outputs such as insensible losses. In the subsequent discussion, we discuss the evolution of these approaches highlighting advantages and disadvantages and refer the reader to the supplement for mathematical details.

A straightforward approach focused on the concept of “mass balance” (46). In this method, the fluid balance of all inputs and outputs is added to TBW in the denominator of the idealized plasma [Na^+^] relationship in Eq. 1, whereas total monocation (Na^+^ and K^+^) input minus output is added to the numerator. Nguyen and colleagues emphasized the use of the empiric Edelman plasma [Na^+^] relationship in Eq. 2 instead of the idealized relationship in Eq. 1 in a modification of this approach (39). The approach is commonly applied retrospectively with input and output volume and composition rigorously measured and accounted over a given time frame in which plasma [Na^+^] changes from an initial to a final value. The initial TBW is estimated using weight-based rules of thumb or anthropometric equations and then the monocation and volume balances along the initial plasma [Na^+^] are used to calculate expected final plasma [Na^+^], which is then compared to the observed final plasma [Na^+^] (46). Deviations between expected and observed final plasma [Na^+^] are presumed to be due to errors in TBW estimation, measurement or accounting errors of inputs and outputs, and limitations of the chosen plasma [Na^+^] relationship (i.e., idealized or empiric Edelman).

Adrogue and colleagues introduced a variant of the tonicity approach, which calculates a change in plasma [Na^+^] associated with each liter of an infusate based on the idealized plasma [Na^+^] relationship (77, 78). Of course, except in an anuric patient with no significant gastrointestinal or cutaneous losses, the approach would predictably be inaccurate as it fails to account for outputs. To account for this shortcoming, these investigators later provided a parallel formula to predict a change in plasma [Na^+^] with each liter of an output (40). However, Barsoum and Levine aptly pointed out that the final change in plasma [Na^+^] is not simply the volume weighted sum of the all of the changes in plasma [Na^+^] per liter of input and output. Therefore, these investigators derived a formula for the change in plasma [Na^+^] based on the idealized plasma [Na^+^] relationship accounting for all input and outputs, which essentially recapitulates the mass balance approach with a mathematical modification (76).

The use of urinary EFWC in the analysis of dysnatremias was introduced by Goldberg (41) and further expanded by Rose (4). Urinary EFWC built upon the concept of urinary free water clearance devised by classic renal physiologists to quantify renal water handling (79), but appropriately removed the contribution of urinary excretion of ineffective osmoles, such as urea. As with the absence of outputs in the original Adrogue infusate approach, urinary EFWC failed to account for inputs and non-renal outputs. Several authors, therefore, extended the concept of electrolyte-free water to both inputs and outputs to generate an EFWB based on the idealized or empiric Edelman relationship (40, 42, 46). These quantities are the volume equivalents over a given time frame of the volumetric rates defined by EFWI, EFWC, and EFWB herein. Halperin and colleagues calculated EFWI and EFWC volumes for each input and output, respectively and then summed these to arrive at an EFWB volume (46). These authors added EFWB volume to TBW in the denominator of Eq. 1 to yield a final plasma [Na^+^], which is quantitatively reasonable but theoretically incorrect, since EFWB, even though expressed as a volume, is a composite of monocation and fluid balances. Nguyen and colleagues derived an expression for a volume denoted as whole body-EFWC (WB-EFWC) based on the empiric Edelman equation using the concepts that volume balance may be separated into isotonic and electrolyte-free water components and that WB-EFWC is zero when plasma [Na^+^] does not change over a given time frame (42). This derivation connected EFWB to monocation and volume balance and the plasma [Na^+^] relationship allowing practitioners to qualitatively connect a gain in electrolyte-free water to a fall in plasma [Na^+^] and *vice versa*. In this work, we further clarify that EFWB, defined as a clearance, is proportional to the rate of change in plasma [Na^+^].

The prospective application to design therapy of any of the described methods raises several additional difficulties beyond limitations in the estimation of TBW and accurate accounting of inputs and outputs. If the entire development of the dysnatremia was observed with accurately measured monocation and fluid balances, then one could develop a treatment regimen which simply reverses these balances. However, this scenario occurs in a small minority of cases observed in routine clinical practice. In most cases, without any prior balance information, the primary difficulty lies in the need to independently define a target monocation or fluid balance to then solve the other balance based on a desired final plasma [Na^+^] or change in plasma [Na^+^] over a given time frame. An infinite number of monocation and fluid balance combinations can yield a desired change in plasma [Na^+^]. Moreover, a final plasma [Na^+^] can be reached with grossly different alterations in total body Na^+^, K^+^, and water, some of which would not reverse the pathogenesis and could detrimentally alter clinically relevant body fluid compartments, particularly ECF volume. Classic approaches arbitrarily assume either monocation balance or fluid balance is zero and then calculate the other. Unfortunately, most dysnatremias do not result from pure water or cation alterations, but rather result from mixed gains or losses of Na^+^, K^+^, and water (28, 64, 65, 80–82). In the Adrogue infusate approach, an infusate is chosen which allows one to calculate a change in plasma [Na^+^], but the actual monocation and volume balances can vary significantly based on which infusate is chosen. We suggest that focusing on a desired change in ECF volume, a clinically critical variable in dysnatremias, allows one to define Na^+^ balance, which along with an empirically determined K^+^ balance, independently delineates a target monocation balance.

An additional issue with designing therapy is spontaneous or treatment-induced alterations in EFWB. Inputs, such as fluid boluses and IV medications, can be prescribed *ad hoc* throughout the course of the day or outputs, such as blood loss or diarrhea, may occur unexpectedly in a complex, critically ill patient. Suppression of ADH with volume resuscitation in hypovolemic hyponatremia, administration of corticosteroids in adrenal insufficiency, or the waning effect of an ADH-stimulating medication may lead to a brisk diuresis significantly raising EFWC (38). Alternatively, administration of normal saline in SIADH may raise urinary Na^+^ excretion and reduce EFWC (83). None of the mathematical approaches can account for unexpected or therapy associated alterations in EFWB. Thus, frequent plasma [Na^+^] measurements and assessment of inputs and outputs, such as urine flow rate and chemistries, along with clinical follow-up are mandatory in the management of dysnatremias to modify the treatment plan after calculations have provided a starting point. Each of these laboratory and clinical reassessments may be accompanied by a recalculation of the rate of change in plasma [Na^+^] with recalibration of therapy.

## Conclusion

Theoretically speaking, the mass balance, Adrogue change in plasma [Na^+^], and EFWB approaches are equivalent as they all draw upon the idealized or empiric Edelman plasma [Na^+^] relationships. However, we believe that the EFWB concept is appealing at the bedside, because it leverages the clinical intuition of non-isotonic gains and losses as primary drivers of dysnatremias and their repair. In this review, we focus attention on EFWB as a clearance or volumetric rate and mathematically connect it to the rate of change in plasma [Na^+^]. The connection between EFWB and the rate of change in plasma [Na^+^] establishes that EFWB is actively regulated to zero in normal physiology to maintain plasma [Na^+^] at its homeostatic set point. Persistently positive or negative EFWB results in hyponatremia or hypernatremia, respectively. The rate of change in plasma [Na^+^] is also clinically relevant as it is targeted by clinicians during therapy to prevent complications associated with overcorrection of dysnatremias (84). The construct of rates also compactly defines a rate of change in ECF volume using the sum of the entire isotonic volume, but only a fraction of the free water, a concept intuitively known by most clinicians. The rate of change in ECF volume then allows one to define a target Na^+^ balance which along with an empirically determined K^+^ balance yields a monocation balance providing a physiologically based technique to clearly define Na^+^, K^+^, and fluid balances to achieve a rate of change in plasma [Na^+^]. Admittedly, the rate of change in plasma [Na^+^] only approximates the “integrated” change in plasma [Na^+^] that occurs over a given time frame. But, given the need to frequently reassess plasma [Na^+^] and input and output volumes and composition and clinical course in the management of complex dysnatremias, the rate of change in plasma [Na^+^] can be used as a moving “snapshot” in time being iteratively calculated and therapy modified as needed without accruing quantitative error.

Taken together, we suggest an approach based on EFWB clearance and rate of change in plasma [Na^+^], but acknowledge that all quantitative approaches are similarly meritorious, given the many theoretical and practical shortcomings described herein. Thus, it is not surprising that these approaches are equally modest in their ability to predict alterations in plasma [Na^+^] (33–37). Despite these limitations, we believe that insight into the EFWB concept gained from this review will allow clinicians to better understand the pathogenesis and management of dysnatremias.

## Author Contributions

SS and GB conceived of and wrote the manuscript.

## Conflict of Interest Statement

The authors declare that the research was conducted in the absence of any commercial or financial relationships that could be construed as a potential conflict of interest.
