# Supplementary material for: Using Electrolyte Free Water Balance to Rationalize and Treat Dysnatremias
Source: Front Med (Lausanne). 2018 Apr 23;5:103. doi: 10.3389/fmed.2018.00103 (PMC5925609; doi:10.3389/fmed.2018.00103)
Supplement: Supplementary file 1 [file data_sheet_1.PDF]

# Supplemental Information

**I. Derivation of dPNa/dt – EFWB and dECFV/dt Relationships**
**II. Additional Illustrative Case of Hypovolemic Hypernatremia**
**III. Mathematical Details for Quantitative Approaches to Dysnatremias**

## I. Derivation of dPNa/dt – EFWB and dECFV/dt Relationships

The starting point for deriving the rate of change in plasma [$Na^+$] relationship was a modified version of the idealized plasma [$Na^+$] relationship (equation 1) (1, 2):

$$P_{[Na+K]} = f_{PW} * \frac{Na_e + K_e - a}{TBW} \qquad \text{(eqn. 1)}$$

where $P_{Na+K}$ represents plasma [$Na^+ + K^+$], $f_{PW}$ is the fraction of plasma that is water, $Na_e$ and $K_e$ are exchangeable $Na^+$ and $K^+$, TBW is total body water, and a is assumed to be a constant related to osmotically inactive, exchangeable $Na^+$ and $K^+$. To examine temporal changes in plasma [$Na^+$], $P_{Na+K}$ is envisioned as a time dependent function with its components also seen as time dependent variables:

$$P_{Na+K}(t) = f_{PW} * \frac{[Na_e + K_e](t) - a}{TBW(t)} \qquad \text{(eqn. 2)}$$

Standard rules of differentiation and assuming $dP_{Na+K}/dt \cong dP_{Na}/dt$ yields the following (3):

$$dP_{Na}/dt = f_{PW} * \frac{d[Na_e + K_e]/dt * TBW(t) - dTBW/dt * ([Na_e + K_e](t) - a)}{[TBW(t)]^2} \qquad \text{(eqn. 3)}$$

If we assume an input with a constant flow rate of $V_I$ and electrolyte concentration of $I_{[Na+K]}$, an output with a constant flow rate of $V_O$ and electrolyte concentration of $O_{[Na+K]}$, and that changes in external cation and volume balances (CB and VB) equal changes in $Na_e$, $K_e$, and TBW, then the derivatives can be expressed as follows:

$$dMCB/dt = V_I * I_{[Na+K]} – V_O * O_{[Na+K]} = d[Na_e + K_e]/dt \quad \text{(eqn. 4)}$$

$$dVB/dt = V_I - V_O = dTBW/dt \quad \text{(eqn. 5)}$$

If we substitute equations 4 and 5 into equation 3 and solve for the rate of change in plasma $[Na^+]$ at time t = 0 with $P_{[Na+K]i}$ and $TBW_i$, then $dP_{Na}/dt$ is delineated as follows:

$$dP_{Na}/dt = f_{PW} * \frac{(V_I * I_{[Na+K]} – V_O * O_{[Na+K]}) – [(V_I – V_O) * P_{[Na+K]i} / f_{PW}]}{TBW_i} \quad \text{(eqn. 6)}$$

First, we let $P_{[Na+K]} / f_{PW} = P[Na+K]_{pw}$. Second, we group all of the $V_I$ and $V_O$ terms in the numerator with one another and factor out $P_{[Na+K]i}$, then the rate of change in plasma $[Na^+]$ is as follows:

$$dP_{Na}/dt = - \frac{[V_I * (1 – I_{[Na+K]}/P_{[Na+K]pwi})] – [V_O * (1 – O_{[Na+K]}/P_{[Na+K]pwi}]}{TBW_i} * P_{[Na+K]i} \quad \text{(eqn. 7)}$$

The second bracketed term in the numerator is recognizable as electrolyte free water clearance (EFWC), while the first term can be considered its intake analogue which we term electrolyte free water intake (EFWI):

$$dP_{Na}/dt = \frac{- (EFWI – EFWC)}{TBW_i} * P_{[Na+K]i} \quad \text{(eqn. 8)}$$

$$EFWC = V_O * [1 – (O_{[Na+K]} / P_{[Na+K]pwi})] = V_O – (V_O*O_{[Na+K]} / P_{[Na+K]pwi}) \quad \text{(eqn. 9)}$$

$$EFWI = V_I * [1 – (I_{[Na+K]} / P_{[Na+K]pwi})] = V_I – (V_I*I_{[Na+K]} / P_{[Na+K]pwi}) \quad \text{(eqn. 10)}$$

The difference in EFWI and EFWC can be defined as electrolyte free water balance (EFWB):

$$EFWB = EFWI – EFWC \quad \text{(eqn. 11)}$$

$$dP_{Na}/dt = \frac{- EFWB}{TBW_i} * P_{[Na+K]i} \quad \text{(eqn. 12)}$$

Alternatively, the terms in equation 7 can be left as is with only $P_{[Na+K]i}$ factored out allowing us to relate dPNa/dt to rates of change in volume balance (dVB/dt) and monovalent cation balance (dMCB/dt) and then relate these to EFWB:

$$dP_{Na}/dt = - \frac{dVB/dt - [(dMCB/dt) / P_{[Na+K]pwi}]}{TBW_i} * P_{[Na+K]i} = - \frac{EFWB}{TBW_i} * P_{[Na+K]i} \quad \text{(eqn. 13)}$$

$$EFWB = dVB/dt - \frac{dMCB/dt}{P_{[Na+K]pwi}} \quad \text{(eqn. 14)}$$

To better guide therapy, we also wanted to relate dMCB/dt and EFWB to changes in extracellular and intracellular volumes (ECFV and ICFV respectively), since these are real volumes we alter with therapy. Notably, as ECFV and ICFV comprise the total body water of an individual, dVB/dt can be written as:

$$dVB/dt = dECFV/dt + dICFV/dt \quad \text{(eqn. 15)}$$

If we consider that ECF and ICF volumes can each be theoretically split further into two hypothetical volumes, one consisting of pure water and another composed of isotonic fluid, one can model changes to either compartment incorporating the ideas of EFWB and monovalent cation balance derived above. In this simplified model, electrolyte free water is added to or removed from ECF and ICF in direct proportion to their fractional contributions to TBW, whereas isotonic changes in $Na^+$ and $K^+$ balance affect the ECF and ICF given their predominance in the respective spaces. Thus, the rate of change in ECF and ICF volume can be expressed as follows:

$$dECFV/ dt = \frac{dNaB/dt}{P_{[Na+K]pwi}} + (f_{ECF} * EFWB) \quad \text{(eqn. 16)}$$

$$dICFV/dt = \frac{dKB/dt}{P_{[Na+K]pwi}} + (f_{ICF} * EFWB) \qquad \text{(eqn. 17)}$$

Here, $(dNaB/dt)/P_{[Na+K]i}$ and $(dKB/dt)/P_{[Na+K]i}$ represent the isotonic volume changes affecting the ECF and ICF respectively, while $f_{ECF}$*(EFWB) and $f_{ICF}$*(EFWB) represent the changes in the ECF and ICF respectively due to changes in pure water balance based on the fractional distribution of water ($f_{ECF}$ and $f_{ICF}$) between the ICF and ECF. Based on these definitions, we note the following straightforward relationships:

$$dMCB/dt = dNaB/dt + dKB/dt \qquad \text{(eqn. 18)}$$

$$f_{ECF} + f_{ICF} = 1 \qquad \text{(eqn. 19)}$$

We recognize many shortcomings exist to this model including, but not limited to: 1) Some exchangeable $Na^+$ distributes into ICF and compartments with altered osmotic activity, 2) A small fraction of exchangeable $K^+$ is found extracellularly, and 3) The accompanying anion may affect the relationship between $Na^+$ balance and changes in ECF volume and $K^+$ balance and changes in ICF volume (1, 4, 5). While these effects may be incorporated into more complicated mathematical models, we wanted to formalize the predominance of $Na^+$ in ECF and $K^+$ in ICF and the difference in distribution between isotonic volume and EFW for simple application at the bedside.

## II. Additional Illustrative Case of Hypovolemic Hypernatremia

An 89 year old gentleman residing in a long term care facility with a past medical history significant for moderate Alzheimer's dementia, BPH with indwelling urinary catheter, and chronic diarrhea (8 weeks duration) of unclear etiology was found to have worsening mental

status over the course of several days with diminished oral intake. Physical exam was remarkable for disorientation, weight of 85 kg, BP of 101/50, flat JVP, dry mucous membranes, and no edema. Labs were notable for a plasma [$Na^+$] of 153 mEq/L, [$K^+$] = 3.0 mEq/L, [$Cl^-$] = 116 mEq/L, [$HCO_3^-$] = 24 mEq/L, BUN = 14 mg/dL and creatinine = 1.2 mg/dL. Urine osmolality was 500 mOsm/kg, urine [$Na^+$] was 20 mEq/L and urine [$K^+$] was 15 mEq/L. Urine flow rate was 400 mL/day. The patient was diagnosed with catheter associated urinary tract infection and started on ceftriaxone in the emergency department. Previous stool studies were notable for multiple negative C. difficile PCRs and negative fecal leukocytes, bacterial stool culture, ova and parasites, and fecal fat staining. Given the patient's ICF and ECF volume contraction, TBW was estimated at 34L (0.4 * 85 kg) with $f_{ECF}$ = 0.33. $P_{[Na+K]}$ = 156 mEq/L, while $P_{[Na+K]pw}$ = 167.7 mEq/kg assuming a normal 93% plasma water. In this case, diarrheal loses were significant and therefore required estimation. The history of chronic diarrhea with negative infectious work-up, negative fecal fat, and persistence in the absence of oral intake suggested a near isotonic, secretory diarrhea. We assumed 1L stool output with stool [$Na^+$] = 100 mEq/L and [$K^+$] = 40 mEq/L. Based on these assumptions, we targeted an ECF expansion of 1L (about 10%) and a drop in plasma [$Na^+$] of 8 mEq/L/day and applied the stepwise approach:

***Step 1: Define EFWB Target Rate***

$$\text{EFWB} = -\ R * \text{TBW} / P_{[Na+K]}$$

$$\text{EFWB} = -\ -\ 8\ \text{mEq/L/day} * 34\ \text{L} / 156\ \text{mEq/L} = 1.74\ \text{L/day}$$

***Step 2: Define $Na^+$ Balance Rate Using ECF Volume and EFWB Target Rates***

$$\text{dECFV/dt} = +\ 1\ \text{L/day}$$

$$\text{dECFV/ dt} = \frac{\text{dNaB/dt}}{P_{[Na+K]pw}} + (f_{ECF} * \text{EFWB}) = +\ 1\ \text{L/day}$$

$$\frac{dNaB/dt}{P_{[Na+K]pw}} = dECFV/dt - 1/3 * EFWB = 1 - [(0.33 * 1.74)] = + 0.42 \text{ L/day}$$

$$dNaB/dt = P_{[Na+K]pw} * 0.42\text{L/day} = 167.7 \text{ mEq/L} * 0.42\text{L/day} = 70.43 \text{ mEq/day}$$

$$dNaB/dt = \text{IV } Na^+ \text{ Input Rate} - \text{Urinary } Na^+ \text{ Excretion} - \text{Fecal } Na^+ \text{ Loss} = 70.43 \text{ mEq/day}$$

$$\mathbf{IV\ Na^+\ Input\ Rate = 70.43\ mEq/day + 8\ mEq/day + 100\ mEq/day = 178.43\ mEq/day}$$

***Step 3: Determine if any $K^+$ Supplementation is Required and Isotonic Volume Rate***

$$dKB/dt = + 40 \text{ mEq/day}$$

$$\frac{dKB/dt}{P_{[Na+K]pw}} = \frac{40 \text{ mEq/d}}{167.7 \text{ mEq/kg}} = 0.24 \text{ L/day}$$

$$dKB/dt = \text{IVF } K^+ \text{ Input Rate} - \text{Urinary } K^+ \text{ Excretion} - \text{Fecal } K^+ \text{ Excretion} = 40 \text{ mEq/day}$$

$$\mathbf{IVF\ K^+\ Input\ Rate = 40\ mEq/day + 6\ mEq/day + 40\ mEq/day = 86\ mEq/day}$$

$$\frac{dMCB/dt}{P_{[Na+K]pw}} = \frac{dNaB/dt}{P_{[Na+K]pw}} + \frac{dKB/dt}{P_{[Na+K]pw}} = 0.42 + 0.24 = 0.66 \text{ L/day}$$

***Step 4: Calculate Volume Balance Rate Based on EFWB Target and Isotonic Volume Rates***

$$dVB/dt = EFWB + \frac{dMCB/dt}{P_{[Na+K]pw}} = 1.74 + 0.66 = 2.4 \text{ L/day}$$

$$dVB/dt = V_{IVF} + V_{oral} + V_{unmeasured} - V_{urine} - V_{fecal} = 2.4 \text{ L/day}$$

$$V_{oral} = 0$$

$$V_{unmeasured} = - 0.5\text{L/day}$$

$$V_{urine} = 0.4 \text{ L/day}$$

$$V_{fecal} = 1 \text{ L/day}$$

$$\mathbf{V_{IVF} = dVB/dt - (V_{oral} - V_{unmeasured}) + V_{urine} + V_{fecal} = 2.4 + 0.5 + 0.4 + 1.0 = 4.3\ L/day}$$

Taken together, we needed to provide 177 mEq $Na^+$ and 86 mEq $K^+$ along with 4.3L of intravenous fluid. To make this clinically feasible, we prescribed 2.3L of 1/2NS + 20 mEq/L KCl (177 mEq $Na^+$, 46 mEq $K^+$) followed by 2L $D_5W$ with 20 mEq/L KCl (40 mEq $K^+$).

Calculation of a TBW deficit is the classic approach to hypernatremia. The TBW deficit is calculated in this case as follows:

$$\text{TBW Deficit} = \text{TBW} * [(\text{Plasma } [Na^+]_f / \text{Plasma } [Na^+]_i) - 1)]$$

$$\text{TBW Deficit} = 34 \text{ L} * [(153 / 145) - 1] = 1.88 \text{ L}$$

Some would stop at this juncture and plan on administering 1.88L of $D_5W$ over the next day. This strategy would only lower plasma [$Na^+$] by 4.5 mEq/L and would allow hypovolemia to persist and probably worsen. The primary reason for the under-correction of plasma [$Na^+$] is the failure to account for on-going losses and may underlie the observation of slow correction rates in hospitalized patients with hypernatremia (6). Thus, many astute clinicians would estimate on-going $Na^+$, $K^+$, and fluid losses and add these expected losses to the calculated TBW deficit:

$$V_{insensible} = 0.5 \text{ L/day}$$

$$V_u = 0.4 \text{ L/day}$$

$$\text{Urinary } Na^+ \text{ Excretion Rate} = 20 \text{ mEq/L} * 0.4 \text{ L/day} = 8 \text{ mEq/day}$$

$$\text{Urinary } K^+ \text{ Excretion Rate} = 15 \text{ mEq/L} * 0.4 \text{ L/day} = 6 \text{ mEq/day}$$

$$V_{fecal} = 1\text{L/day}$$

$$\text{Fecal } Na^+ \text{ Excretion Rate} = 100 \text{ mEq/L} * 1 \text{ L/day} = (100 \text{ mEq/day})$$

$$\text{Fecal } K^+ \text{ Excretion Rate} = 40 \text{ mEq/L} * 1 \text{ L/day} = (40 \text{ mEq/day})$$

$$\textbf{Total Volume = TBW Deficit + } \mathbf{V_{insensible} + V_{urine} + V_{fecal}} \textbf{ = 1.88 + 0.5 + 0.4 + (1) = 3.8 L/day}$$

$$\mathbf{Na^+} \textbf{ Requirement = 108 mEq/day}$$

$$\mathbf{K^+} \textbf{ Requirement = } \mathbf{K^+} \textbf{ Loss + } \mathbf{K^+} \textbf{ Supplementation = 46 mEq + 40 mEq = 86 mEq/day}$$

Thus, we could provide 1.4 L of ½ NS + 25 mEq/L KCl (108 mEq Na+, 35 mEq $K^+$) followed by 2.4L D5W + 20 mEq/L KCl (48 mEq $K^+$) to closely approximate the calculated requirements. The resulting fall in plasma [$Na^+$] is predicted to be 7.1 mEq/L, but ECF volume expansion would only be about 0.56L, which is short of our desired target. Despite carefully accounting for on-going losses, the TBW deficit approach provides for reasonable plasma [$Na^+$] correction, but falls short on ECF volume resuscitation. It is evident that the TBW deficit and our approach both require a similar number of calculations and estimates of on-going $Na^+$, $K^+$, and fluid losses. Despite the equivalent complexity, the TBW deficit approach potentially prolongs hypovolemia, while our approach explicitly integrates ECF volume repair into the therapeutic regimen to correct both ECF volume and plasma [$Na^+$].

## III. Mathematical Details for Quantitative Approaches to Dysnatremias

All proposed approaches to dysnatrmias are based on the idealized or empiric Edelman equations. As noted in the manuscript, we will use a modified version of the idealized plasma [Na+] relationship for clarity, but note that analogous equations may be derived using the empiric Edelman equation as done by Nguyen and colleagues (7).

### A. "Mass Balance" Approach

The mass balance approach simply suggests that the final plasma [Na+K] ($P_{[Na+K]f}$) concentration reached reflects the external monocation (ΔMCB) and fluid (ΔVB) added to the initial exchangeable cation (($Na_e + K_e)_i$) and TBW ($TBW_i$) respectively:

$$P_{[Na+K]i} = f_{PW} * \frac{(Na_e + K_e)_i - a}{TBW_i} \qquad \text{(eqn. 20)}$$

$$P_{[Na+K]f} = f_{PW} * \frac{(Na_e + K_e)_i - a + \Delta MCB}{TBW_i + \Delta VB} \quad \text{(eqn. 21)}$$

Subtracting initial $P_{[Na+K]}$ from final $P_{[Na+K]}$ yields the change in plasma [Na+K]:

$$\Delta P_{[Na+K]} = \text{Final } P_{[Na+K]} - \text{Initial } P_{[Na+K]} \quad \text{(eqn. 22)}$$

$$\Delta P_{[Na+K]} = \frac{(f_{pw} * TBW_i * \Delta MCB) - [f_{pw} * \Delta VB * ((Na_e + K_e)_i - a))]}{TBW_i * ( TBW_i + \Delta VB)} \quad \text{(eqn. 23)}$$

$$(Na_e + K_e)_i - a = TBW_i * P_{[Na+K]i} / f_{pw} \quad \text{(eqn. 24)}$$

$$\Delta P_{[Na+K]} \cong \Delta \text{Plasma } [Na^+] \quad \text{(eqn. 25)}$$

$$\Delta \text{Plasma } [Na^+] = \frac{(f_{pw} * \Delta MCB) - (\Delta VB * P_{[Na+K]i})}{TBW_i + \Delta VB} \quad \text{(eqn. 26)}$$

Using equation 26, one can calculate the change in plasma [$Na^+$] expected to occur over a time frame based on the initial plasma [Na+K] and the monocation (ΔMCB) and volume (ΔVB) balances which occurred over that same time frame assuming $f_{pw}$ and initial TBW ($TBW_i$) can be estimated.

**B. Adrogue Variant of the Mass Balance Approach**

Adrogue and colleagues suggest examining the change in plasma [$Na^+$] that would occur with 1L of a given input or output (8). For a 1L input or output, the volume balance would then be +1L or -1L respectively and the monocation balance for the 1L input ($\Delta MCB_{in}$) or 1L output ($\Delta MCB_{out}$) would be as follows:

$$\Delta MCB_{in} = \Delta VB_{in} * I_{[Na+K]} = I_{[Na+K]} \quad \text{(eqn. 27)}$$

$$\Delta MCB_{out} = \Delta VB_{out} * O_{[Na+K]} = - O_{[Na+K]} \quad \text{(eqn. 28)}$$

If $f_{pw}$ is ignored and assumed to be 1, then the following equations for change in plasma $[Na^+]$ may be derived using equations 26-28:

$$\Delta \text{Plasma } [Na^+]_{1Lin} = \frac{I_{[Na+K]} - P_{[Na+K]i}}{TBW_i + 1} \qquad \text{(eqn. 29)}$$

$$\Delta \text{Plasma } [Na^+]_{1Lout} = \frac{P_{[Na+K]i} - O_{[Na+K]}}{TBW_i - 1} \qquad \text{(eqn. 30)}$$

Intuitively, if one wants to calculate the change in plasma $[Na^+]$ due to an input or output that is not 1L, one might consider the following equations:

$$\Delta \text{Plasma } [Na^+]_{in} = \Delta VB_{in} * \Delta \text{Plasma } [Na^+]_{1Lin} \qquad \text{(eqn. 31)}$$

$$\Delta \text{Plasma } [Na^+]_{out} = - \Delta VB_{out} * \Delta \text{Plasma } [Na^+]_{1Lout} \qquad \text{(eqn. 32)}$$

As pointed out by others (9), mathematically, equations 31 and 32 are not correct as they do not equal the value obtained using the parent equation 26 even if $f_{pw}$ is neglected as the dominators differ with the result of equation 26 shown on the right side of the inequality:

$$\Delta VB_{in} * \Delta \text{Plasma } [Na^+]_{1Lin} \neq \frac{\Delta VB_{in} * (I_{[Na+K]} - P_{[Na+K]i})}{TBW + \Delta VB_{in}} \qquad \text{(eqn. 33)}$$

$$- \Delta VB_{out} * \Delta \text{Plasma } [Na^+]_{1Lout} \neq \frac{\Delta VB_{out} * (O_{[Na+K]} - P_{[Na+K]i})}{TBW + \Delta VB_{out}} \qquad \text{(eqn. 34)}$$

The error is small quantitatively as TBW dominates over volume balance ($\Delta VB$) in the denominator and the estimation of initial TBW is itself significantly prone to error.

## C. Electrolyte Free Water Balance Approach

Using equation 26 and factoring out $P_{[Na+K]i}$ from the numerator yields an equation relating the change in plasma $[Na^+]$ over a time frame to EFWB expressed as a volume over the same time frame (ΔEFWB):

$$\Delta Plasma\ [Na^+] = \frac{\dfrac{\Delta MCB}{P_{[Na+K]pwi}} - \Delta VB}{TBW_i + \Delta VB} * P_{[Na+K]i} \qquad \text{(eqn. 35)}$$

$$\Delta EFWB = \Delta VB - \frac{\Delta MCB}{P_{[Na+K]pwi}} \qquad \text{(eqn. 36)}$$

$$\Delta Plasma\ [Na^+] = \frac{-\Delta EFWB}{TBW_i + \Delta VB} * P_{[Na+K]i} \qquad \text{(eqn. 37)}$$

Thus, a change in plasma $[Na^+]$ over a time frame can be calculated using equation 37 with the electrolyte free water balance and the volume balance expressed as volumes over the same time frame. ΔEFWB is similar to the WB-EFWC quantity defined by Nguyen and colleagues except that WB-EFWC is derived from the empiric Edelman equation rather than the idealized plasma $[Na^+]$ relationship used here (10). The fractional change in plasma $[Na^+]$ is proportional to ΔEFWB scaled to the final TBW (i.e. $TBW_i + \Delta VB$) with gains of EFWB leading to a decrease in plasma $[Na^+]$ and vice versa. Neutral EFWB equal to zero leads to no change in plasma $[Na^+]$ as expected.

Some authors have suggested an alternate way of using ΔEFWB (or ΔEFWC) to calculate Δplasma [$Na^+$] where ΔEFWB is added to the initial TBW to calculate the final plasma [$Na^+$] (11, 12):

$$P_{[Na+K]f} = f_{PW} * \frac{(Na_e + K_e)_i - a}{TBW_i + \Delta EFWB} \qquad \text{(eqn. 38)}$$

This technically leads to a slightly different result from the actual change in plasma [$Na^+$] which is similar but not the same as the parent equation 37 shown on the right:

$$\Delta \text{Plasma } [Na^+] = \frac{-\Delta EFWB}{TBW_i + \Delta EFWB} * P_{[Na+K]i} \neq \frac{-\Delta EFWB}{TBW_i + \Delta VB} * P_{[Na+K]i} \qquad \text{(eqn. 39)}$$

## References

1. Bhave G, Neilson EG. Body fluid dynamics: back to the future. *J Am Soc Nephrol* (2011) 22(12):2166-81. doi: 10.1681/ASN.2011080865. PubMed PMID: 22034644; PubMed Central PMCID: PMC4096826.
2. Maffly RH, Edelman IS. The role of sodium, potassium and water in the hypo-osmotic states of heart failure. *Progress in cardiovascular diseases* (1961) 4:88-104. PubMed PMID: 13765147.
3. Thomas GB, Finney RL. *Calculus and analytic geometry*. 8th ed. Reading, Mass.: Addison-Wesley (1992).
4. Gowrishankar M, Chen CB, Mallie JP, Halperin ML. What is the impact of potassium excretion on the intracellular fluid volume: importance of urine anions. *Kidney Int* (1996) 50(5):1490-5. PubMed PMID: 8914014.
5. Kurtz TW, Al-Bander HA, Morris RC, Jr. "Salt-sensitive" essential hypertension in men. Is the sodium ion alone important? *N Engl J Med* (1987) 317(17):1043-8. Epub 1987/10/22. doi: 10.1056/NEJM198710223171702. PubMed PMID: 3309653.
6. Alshayeb HM, Showkat A, Babar F, Mangold T, Wall BM. Severe hypernatremia correction rate and mortality in hospitalized patients. *The American journal of the medical sciences* (2011) 341(5):356-60. doi: 10.1097/MAJ.0b013e31820a3a90. PubMed PMID: 21358313.
7. Kurtz I, Nguyen MK. Evolving concepts in the quantitative analysis of the determinants of the plasma water sodium concentration and the pathophysiology and treatment of the dysnatremias. *Kidney Int* (2005) 68(5):1982-93. Epub 2005/10/14. doi: KID652 [pii]10.1111/j.1523-1755.2005.00652.x. PubMed PMID: 16221198.
8. Adrogue HJ, Madias NE. The challenge of hyponatremia. *J Am Soc Nephrol* (2012) 23(7):1140-8. doi: 10.1681/ASN.2012020128. PubMed PMID: 22626822.
9. Barsoum NR, Levine BS. Current prescriptions for the correction of hyponatraemia and hypernatraemia: are they too simple? *Nephrol Dial Transplant* (2002) 17(7):1176-80. PubMed PMID: 12105238.
10. Nguyen MK, Kurtz I. Whole-body electrolyte-free water clearance: derivation and clinical utility in analyzing the pathogenesis of the dysnatremias. *Clinical and experimental nephrology* (2006) 10(1):19-24. doi: 10.1007/s10157-005-0395-2. PubMed PMID: 16544174.
11. Carlotti AP, Bohn D, Mallie JP, Halperin ML. Tonicity balance, and not electrolyte-free water calculations, more accurately guides therapy for acute changes in natremia. *Intensive Care Med* (2001) 27(5):921-4. PubMed PMID: 11430551.
12. Lindner G, Schwarz C, Kneidinger N, Kramer L, Oberbauer R, Druml W. Can we really predict the change in serum sodium levels? An analysis of currently proposed formulae in hypernatraemic patients. *Nephrol Dial Transplant* (2008) 23(11):3501-8. doi: 10.1093/ndt/gfn476. PubMed PMID: 18723567.
